# Radioactive Cobalt(II) Removal from Aqueous Solutions Using a Reusable Nanocomposite: Kinetic, Isotherms, and Mechanistic Study

**DOI:** 10.3390/ijerph14121453

**Published:** 2017-11-24

**Authors:** Xiaotao Zhang, Ximing Wang, Zhangjing Chen

**Affiliations:** 1 College of Science, Inner Mongolia Agricultural University, Hohhot 010018, China; lianzixiaotao@163.com; 2 College of Material Science and Art Design, Inner Mongolia Agricultural University, Hohhot 010018, China; 3 Department of Sustainable Biomaterials Virginia Tech University, Blacksburg, VA 24061, USA; chengo@vt.edu

**Keywords:** nanocomposite, cobalt(II), adsorption, desorption, kinetic, isotherms

## Abstract

A lignocellulose/montmorillonite (LMT) nanocomposite was prepared as a reusable adsorbent for cobalt(II) ions, and characterized by nitrogen (N_2_) adsorption/desorption isotherm, X-ray Diffraction (XRD), Scanning Electron Microscope (SEM), Transmission Electron Microscopy (TEM), and Fourier Transform Infrared Spectroscopy (FTIR). LMT exhibited efficient adsorption of cobalt ions (Co(II)), and the adsorbed Co(II) was readily desorbed by nitric acid (HNO_3_). All parameters affecting the adsorption and/or desorption of Co(II), including initial Co(II) concentration, pH value, temperature, HNO_3_ concentration, and time, were optimized. The kinetic data analysis showed that the adsorption followed the pseudo-second-order kinetic model and fit well into the Langmuir isotherm equation. Notably, the nanocomposite can be used four times without significantly losing adsorbent capability. The Energy-Dispersive X-ray (EDX) and FTIR spectra analysis also revealed that the adsorption mechanism may be mainly a chemical adsorption dominated process.

## 1. Introduction

Industrial wastewater contains various toxic heavy metals that pose significant health risks if they enter the human food chain [1,2]. Cobalt, a toxic element, exits in the wastewater from nuclear power plants as well as from the mining, metallurgical, electroplating, paints, pigments, and electronic industries. The radionuclide ^60^Co(II) presents one of the most serious problems affecting the environment due to its long half-life of 5.27 years. High levels of cobalt may cause paralysis, diarrhea, low blood pressure, lung irritation, and bone defects in humans [3]. Therefore, removal of Co(II) from industrial effluents is crucial. Several different methods exist to remove Co(II), including chemical precipitation, ion-exchange, coagulation, flocculation, and reverse osmosis [4,5,6,7]; unfortunately, most of these methods are expensive and/or environmentally unfriendly. Adsorption has been emerging as an effective technique for removing Co(II) and has many advantages, such as being high efficiency, environmentally friendly, and cost-effective [8,9].

Lignocellulose (LC), a natural renewable polymer, exists widely in plants and is primarily composed of cellulose, hemicellulose, and lignin [10,11]. LC has been considered a possible adsorbent for the removal of heavy metal ions from wastewater [12,13,14]. However, extensive use of LC as an adsorbent has been largely hindered by its polydispersity property and amorphous structure.

On the other hand, montmorillonite (MT) is a type of silicate mineral with a nanolamellar structure, and has a very limited adsorption capacity of heavy metal ions due to low affinity, swelling, and dispersion in water [15,16,17,18,19,20]. To improve the adsorption capacity, MT is often fabricated into a nanocomposite that is an excellent metal adsorbent [21,22].

Bunhu and Tichagwa [23] prepared a lignocellulose–montmorillonite composite, and investigated its adsorption of methyl orange, lead (Pb^2+^), and cadmium (Cd^2+^). Polymer/clay nanocomposites have become a promising alternative for the expansion of industrial and economic activities and the satisfaction of increasingly stringent environmental conditions [24,25,26,27,28]. As a part of our work on the lignocellulose/montmorillonite (LMT) nanocomposite, we systematically characterized the LMT nanocomposite through N_2_ adsorption/desorption isotherm, X-ray Diffraction (XRD), Scanning Electron Microscope (SEM), Transmission Electron Microscopy (TEM), and Fourier Transform Infrared Spectroscopy (FTIR), and performed a detailed study on its adsorption and desorption of Co(II). To focus on ecosystem stability and public health, this study was completed on the adsorption of ^59^Co(II). To our knowledge, no studies have been completed on the adsorption capacity of Co(II) using a LMT nanocomposite. Hence, a reusable LMT nanocomposite was synthesized in this study. All possible parameters affecting adsorption and/or desorption of Co(II), such as initial Co(II) concentration, pH value, temperature, HNO_3_ concentration, and time, were optimized. Then, the LMT nanocomposite absorption procedure was kinetically analyzed, and the reusability of LMT nanocomposite was systematically tested. The prepared LMT nanocomposite showed potential for the adsorption for Co(II) for removal from aqueous solutions.

## 2. Experiments

### 2.1. Materials

LC (SAM-100) was purchased from Beijing Huaduo Biotech Ltd., Beijing, China. MT (cation exchange capacity (CEC) = 100 meq/100 g) was purchased from Zhejiang Feng Hong Clay Chemical Co., Huzhou, China, was washed by deionized water and dried overnight at 70 °C, milled, and sieved to a 200-mesh particles size. Cobalt(II) nitrate hexahydrate (Co(NO_3_)_2_·6H_2_O) was purchased from Shanghai Jinshan Chemical Co., Shanghai, China. All other chemicals and reagents used in the work were of analytical grade and used without further purification. All solutions were prepared by using deionized water.

### 2.2. Reagents

Aqueous stock Co(II) solution (1 mol/L) was prepared by using Co(NO_3_)_2_·6H_2_O, and diluted to desired concentrations (from 0.0015 to 0.0060 mol/L). The pH value of each Co(II) solution was adjusted by using either hydrochloric acid (HCl, 0.1 mol/L) or sodium hydroxide (NaOH, 0.1 mol/L) solutions. All solutions were freshly made for each experiment. The distribution coefficients of ^59^Co(II) and the ratio of the concentration of ^59^Co(II) adsorbed to that in solution was 3.7 × 10^2^ Bq/L.

### 2.3. Synthesis of LMT Nanocomposite

The LMT nanocomposite was prepared as follows: a certain amount of LC was added into aqueous NaOH solution (ω = 0.20) with 1:30 ratio of weight of LC (g) to volume of NaOH solution. The mixture was stirred at room temperature until a uniform LC suspension was formed. Then, the LC-NaOH mixture was added into a suspension of MT (1.0 g in 30 mL of distilled deionized water), and stirred at room temperature for 0.5 h. The resulting mixture was heated at 60 °C and stirred for 6 h and centrifuged, and supernatant was separated, and pH value was adjusted to 7.0 by using a pH meter (PB-10, Shanghai Youyi Instrument Co., Ltd., Shanghai, China). Finally, the supernatant was completely dried under vacuum at 105 °C for 5 h. All samples were ground and sieved to a 200-mesh particle size, and stored in an airtight plastic container until testing.

### 2.4. Adsorption Experiments

LMT nanocomposite (0.1000 g) was accurately weighed and added into 50 mL of Co(II) solution. The suspension was stirred at a uniform speed of 120 rpm in a thermostatic shaker (SHA-C, Shanghai Yiheng Scientific Instrument Co., Ltd., Shanghai, China). During the experimental procedure, the pH value of each Co(II) solution was adjusted to a constant value. When the adsorption equilibrium was reached, the mixtures were centrifuged at 6000 rpm for 5 min. Then, the initial and final concentrations of Co(II) were determined using a double beam ultraviolet (UV)-visible spectrophotometer (TU-1901, Beijing Purkinje General Instrument Co., Ltd., Beijing, China) [29]. The adsorption experiments were performed on different Co(II) initial concentrations, pH values, adsorption temperatures, and adsorption times. To minimize experimental errors, all data reported are average values of three independent tests. Adsorption capacity of the LMT nanocomposite was calculated according to the following mass–balance relationship [30]:

(1) $$q_{t,1}=\frac{\left(C_{0}-C_{t,1}\right)V_{1}\times 58.93}{m_{1}}$$

where *q_t_*_,1_ (mg/g) is the capacity of adsorption at time *t* (min); *C*_0_ and *C_t_*_,1_ (mol/L) are the Co(II) initial and final concentrations at time *t* (min), respectively; *V*_1_ (mL) is the volume of Co(II) solution; and *m*_1_ (g) is the mass of adsorbent. For calculating *q_t_*_,1_, we assumed no loss of Co(II) ions occurred in all experimental procedures.

### 2.5. Desorption and Regeneration Experiments

The Co(II)-loaded LMT nanocomposite (0.1000 g) was accurately weighted, transferred into 0.5 M different 50 mL desorption eluents, and put into an ultrasonic cleaning machine (KS-300EI, Qindao Shengzhong Instrument Co., Ltd., Qingdao, China). When the desorption equilibrium was reached, the suspension was centrifuged, and the concentration of the desorbed Co(II) was determined using the same method as in the adsorption experiment. The effects of different desorption eluents and concentration, desorption temperatures, and ultrasonic desorption times were studied. Similarly, desorption experiments were repeated three times, and reproducibility was within ±3%. The desorption capacity of the Co(II)-loaded LMT nanocomposite was calculated according to the following equation [31,32]:

(2) $$q_{t,2}=\frac{C_{t,2}V_{2}\times 58.93}{m_{2}}$$

where *q_t_*_,2_ (mg/g) is the desorption amount at time *t* (min); *C_t_*_,2_ (mol/L) is the concentration of Co(II) in HNO_3_ solution at time *t* (min); *V*_2_ (mL) is the volume of HNO_3_ solution; and *m*_2_ (g) is final mass of the adsorbent after releasing Co(II).

To investigate the reusability of the LMT nanocomposite, repeated adsorption–desorption experiments were performed. The LMT nanocomposite, after the first batch reaction, was washed with 15 mL distilled deionized water twice to remove the remaining acid, and dried in a vacuum at 70 °C for the next adsorption of Co(II). The regenerated the LMT nanocomposite was used in four consecutive cycles of adsorption–desorption experiments. Meanwhile, the pH of the waste solution containing Co(II) ions was adjusted to a value greater than 10.0 by using 0.1 M NaOH solution, and was completely converted into cobalt(II) hydroxide (Co(OH)_2_) precipitation, based on the solubility product constant (*K*_sp_) for Co(OH)_2_ being 5.92 × 10^−15^ (pH = 9.36). The solvent was heated for evaporation. Then, Co(OH)_2_ precipitation was concentrated, separated, precipitated, and loaded into an closed container, diluted, and used for further experiments.

### 2.6. Characterization

The Brunauer–Emmett–Teller (BET) surface area of the LMT was determined from nitrogen adsorption–desorption isotherms measured at −196 °C using a surface area analyzer (Micromeritics ASAP 2020, Norcross, GA, USA). The pore structure of the LMT was assessed from the isotherms according to conventional procedures. The surface area (*S*_BET_) and total pore volume (*V*_tot_) were determined according to the manufacturer’s software. The micropore surface area (*S*_mic_), mesopore surface area (*S*_meso_), and the mesopore volume (*V*_meso_) were evaluated using the t-plot method. The density functional theory pore size distribution of the LMT was obtained using the Autosorb software package with medium regularization. FTIR spectra were recorded in KBr pellets by FTIR spectrophotometer (Thermo Nicolet, NEXUS, TM, Waltham, MA, USA). X-ray Diffraction (XRD) analysis of the powdered samples was completed using an X-ray power diffractometer with a copper (Cu) anode (PAN Alytical Co. X’pert PRO, Almeloo, The Netherlands), running at 40 kV and 30 mA, scanning from 4° to 18° at 3°/min. Morphological changes and surface analysis of the samples were recorded by scanning electron microscopy with energy dispersive X-ray spectroscopy (SEM-EDX) (HITACHI S-4800, Toyko, Japan). TEM image analysis of the samples was completed with a TEM (JEM-2010, Tokyo, Japan) at 200 kV.

## 3. Results and Discussion

### 3.1. Surface Area and Pore Volume

The adsorptive capacity of the LMT nanocomposite is related to its specific surface and pore volume. The textural parameters of MT, LMT and Co(II)-loaded LMT in Table 1 show that the surface area and total pore volume of LMT nanocomposite were higher than those of MT, at 245.1 m^2^/g and 1.692 cm^3^/g, respectively. After adsorption Co(II) ions, the surface area and total pore volume of Co(II)-loaded LMT were dropped, at 112.3 m^2^/g and 1.117 cm^3^/g, respectively. Especially, the most obvious decrease is the BET specific surface area reduced from 73.4% to 41.1% and pore volume reduced from 69.4% to 30.6% for the mesopores. These results imply that the contribution of the mesopores was dominant and that the porous structure of LMT was well developed. Based on the data in Table 1, LMT can allow the formation of adequate activated sites and functional groups for electrostatic attraction, coordination, and complexation with Co(II) ions.

### 3.2. FTIR Analysis of LC, MT, and LMT

**Figure 1:**
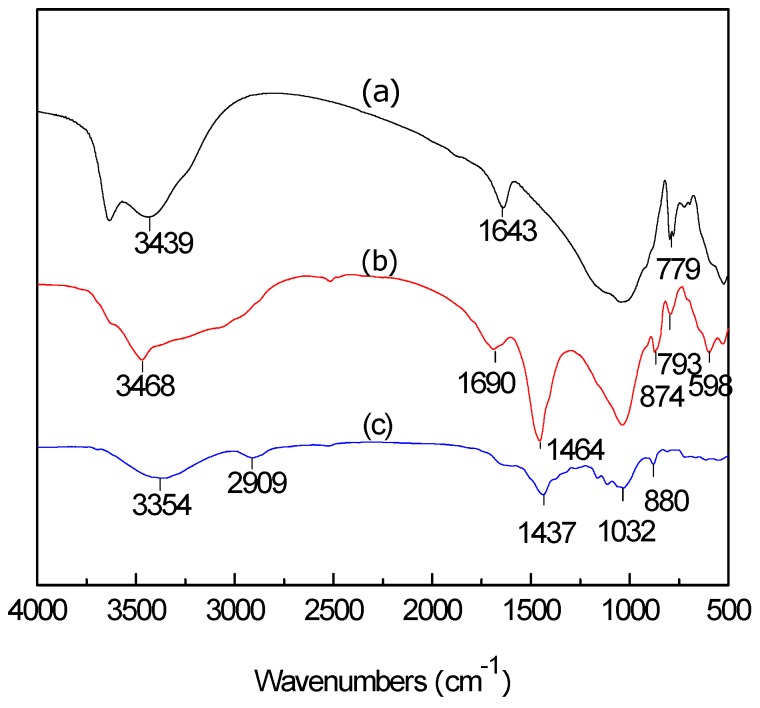
Fourier Transform Infrared Spectroscopy (FTIR) spectra of: (**a**) purified montmorillonite (MT); (**b**) lignocellulose/montmorillonite (LMT); and (**c**) lignocellulose (LC).

FTIR spectra of purified MT, LMT, and LC are shown in Figure 1. The adsorption band at 3439 cm^−1^ (Figure 1a) shifted to the higher wave number 3468 cm^−1^ (Figure 1b), suggesting the vibration band in LC, due to oxygen-hydrogen bond (O–H) stretching, overlapped with the band of MT (–OH stretching vibration of H_2_O). The adsorption band of C–H stretching at 2909 cm^−1^ (Figure 1c) almost disappeared in the nanocomposite (Figure 1b). The characteristic adsorption band of MT at 1643 cm^−1^ (Figure 1a) moved to 1690 cm^−1^ (Figure 1b). Simultaneously, the intensity of this adsorption band decreased, which indicated the –CO group stretching vibration of LC overlapped with the –OH bending vibration of water (H_2_O) in MT. In addition, the band at 1437 cm^−1^, due to C–H bending on methyl and methylene (Figure 1c), was observed and the intensity increased to 1464 cm^−1^ (Figure 1b). The adsorption band at 1043 cm^−1^, caused by the Si–O stretching vibration (Figure 1a), and the adsorption bands at 1139 cm^−1^, 1032 cm^−1^ (for C–O–C and C–O stretching vibration in Figure 1c) in LC weakened, and even disappeared in the nanocomposite (Figure 1b). FTIR spectra comparison showed that MT and LC were completely intercalated, which may have an important influence on the adsorption properties of the nanocomposite.

### 3.3. XRD Analysis of LMT

**Figure 2:**
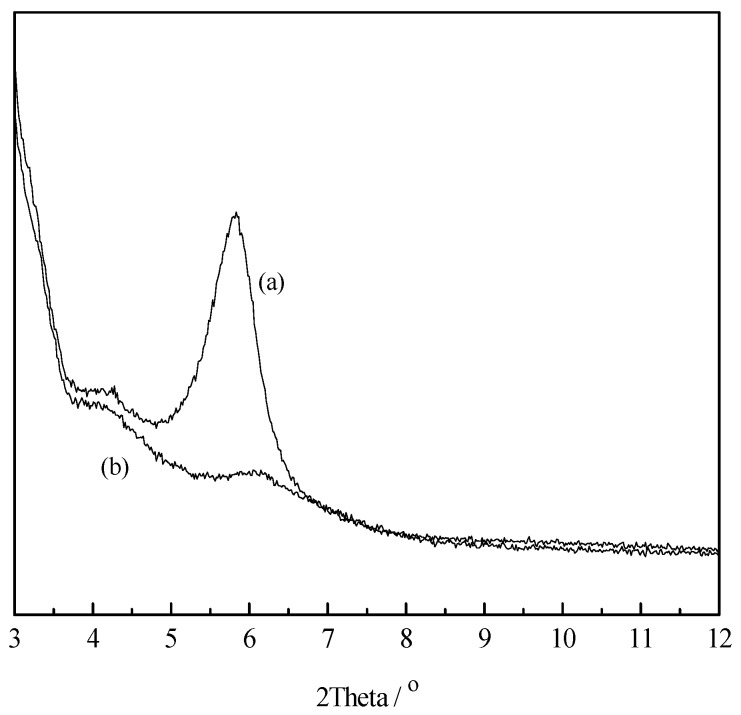
X-ray Diffraction (XRD) patterns of: (**a**) MT; and (**b**) LMT nanocomposite.

X-ray Diffraction (XRD) was an effective way to prove the intercalation of MT and LC. XRD spectra of MT and LMT nanocomposite are shown in Figure 2. The purified MT (Figure 2a) showed a typical diffraction peak at 5.83°, according to the Bragg equation, 2dsinθ = k*λI*, where k = 1, 2, 3, …, corresponding to a basal spacing of 1.52 nm. After intercalation with LC (Figure 2b), the movement of the typical diffraction peak of MT shifted to a lower angle (2θ > 5.83°), indicating the formation of an intercalated nanostructure.

### 3.4. SEM Image Analysis of LMT

**Figure 3:**
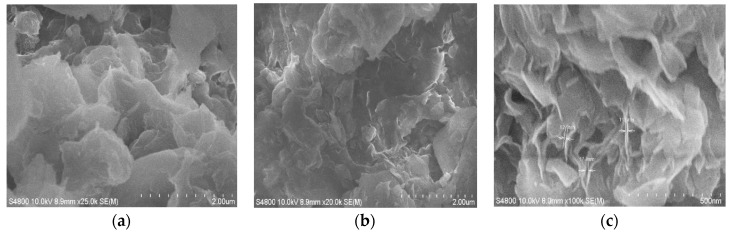
Scanning Electron Microscope (SEM) images of: (**a**) purified MT; and (**b**,**c**) LMT.

The morphologies of purified MT and LMT are shown in Figure 3. Whereas the purified MT showed small particles and a nonporous surface (Figure 3a), the intercalation of LC into MT resulted in large particles and a coarse porous surface (Figure 3b,c), which eventually increased the contact areas for the adsorption of Co(II) ions. In Figure 3c, almost all of the LC was intercalated into the MT interlayer by destroying its crystalline structure, forming intercalated LMT nanocomposite.

### 3.5. TEM Image Analysis of LMT

**Figure 4:**
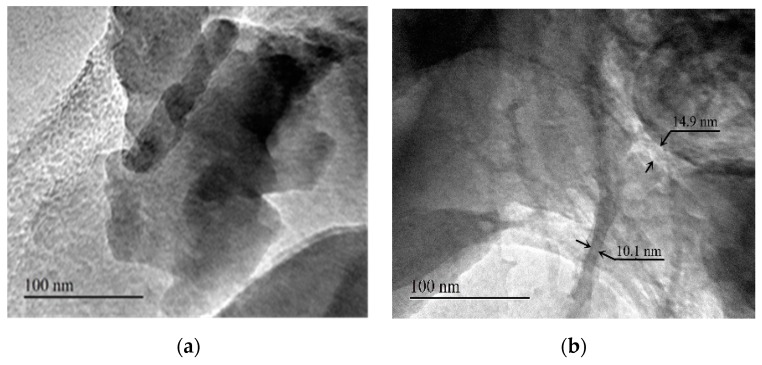
Transmission Electron Microscopy (TEM) images of: (**a**) purified MT; and (**b**) LMT.

TEM images of purified MT and LMT are shown in Figure 4. Compared to MT (Figure 4a), stacks of multilayers of LMT (Figure 4b) became thin and dispersive, indicating that the dispersion of the MT nano-platelets was achieved. Based on the above analytical support from FTIR, XRD, SEM, and TEM, we concluded that the LC was intercalated into the MT interlayer by destroying its crystalline structure.

### 3.6. Effect of Initial Co(II) Concentration on Adsorption

**Figure 5:**
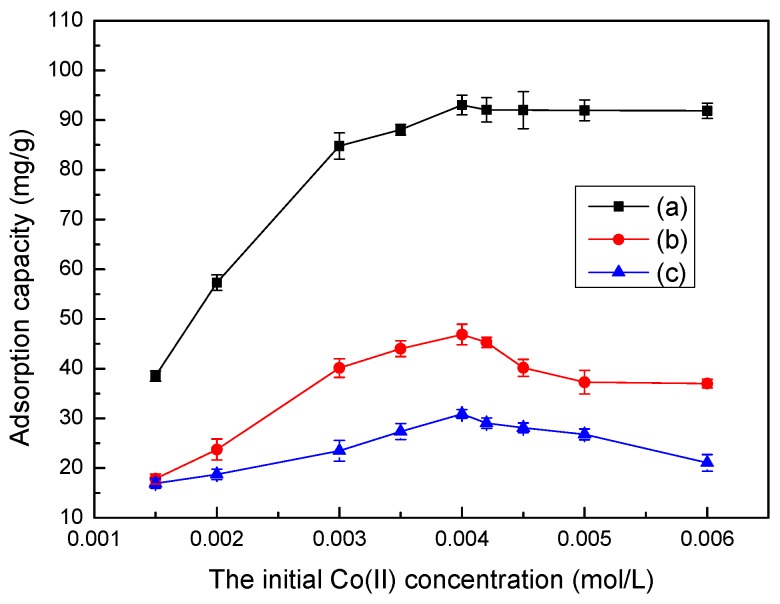
Effect of the initial cobalt ion (Co(II)) concentration on the adsorption capacity of LMT (**a**), MT (**b**), and LC (**c**). Adsorbent: 0.1000 g; pH: 5.6; temperature: 75 °C; time: 60 min.

The initial Co(II) concentration is an important driving force for overcoming mass transfer resistance of Co(II) between the aqueous and solid phases. The effects of different initial Co(II) concentrations on the adsorption capacity of LC, MT, and LMT nanocomposite are shown in Figure 5. The LMT nanocomposite has a much higher adsorption capacity to Co(II) than its parent components, LC and MT. The LMT nanocomposite adsorption capacity of Co(II) is dramatically enhanced with an increase in Co(II) concentration, and then remained constant at higher Co(II) concentrations. The similar trend between the adsorption capacity and initial Co(II) concentration was previously observed on bone char [33]. This phenomenon may be attributed the LMT nanocomposite creating many more adsorption pockets than the parent components. When the Co(II) concentration was further increased, the adsorption capacity remained nearly stable due to the saturation of active adsorption sites. In Figure 5, the highest adsorption capacity of LMT was seen at 0.004 mol/L, and the initial concentration of 0.004 mol/L was used in the following experiments.

### 3.7. Effect of pH Values on Adsorption

**Figure 6:**
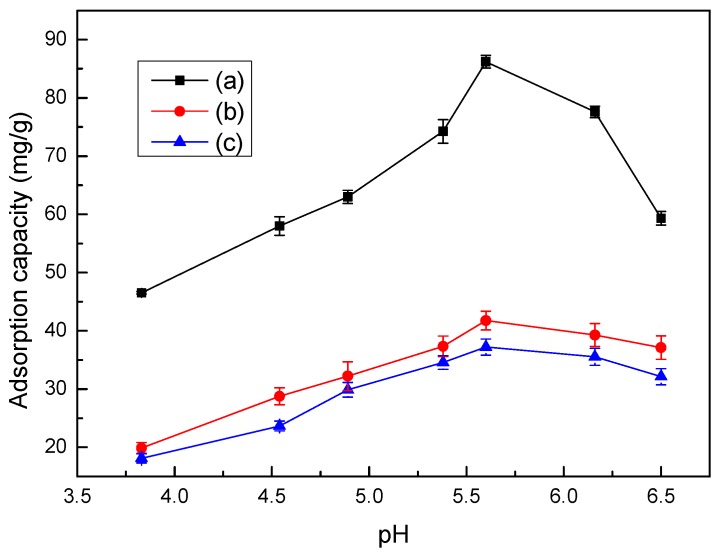
Effect of pH on adsorption capacity of LMT (**a**), MT (**b**), and LC (**c**). Adsorbent: 0.1000 g; initial Co(II) concentration: 0.0040 mol/L; temperature: 75 °C; and time: 60 min.

The pH value is another important factor affecting adsorption of Co(II) on adsorbents, including LMT nanocomposite, LC, and MT. The maximum pH value of the Co(II) solution must avoid the formation of hydrolyzed species. The precipitation was calculated using the concentration of Co(II) and the solubility product constant (*K*_sp_) for Co(OH)_2_ of 5.92 × 10^−15^, and the adsorption pH value should be less than 9.36. The relationship between pH value and adsorption capacity are shown in Figure 6. From Figure 6, the trend on adsorption capacity of LMT of Co(II) exhibited an increase at first, followed by a decrease with increasing pH. When the pH was 5.6, the adsorption capacity reached the maximum amount of 93.02 mg/g. The reason for this is when the pH was less than 5.6, the main reactive functional groups in LMT were –COOH, –OH, and –C=O. Co(II) sorption through the exchange of ions was favorable at low pH values, especially when the sorption rate was largely controlled by ion exchange. As the pH increased, the anion group concentration (–COO^−^) increased, and the coordination and chelation ability of Co(II) with LMT gradually increased. However, when the pH was higher than 5.6, Co(II) could react with a basic pH regulator, which resulted in facile hydroxide complexation or precipitation, and therefore caused a reduction in adsorption capacity [34]. We determined that the optimum pH for adsorption was 5.6.

### 3.8. Effect of Temperature on Adsorption

**Figure 7:**
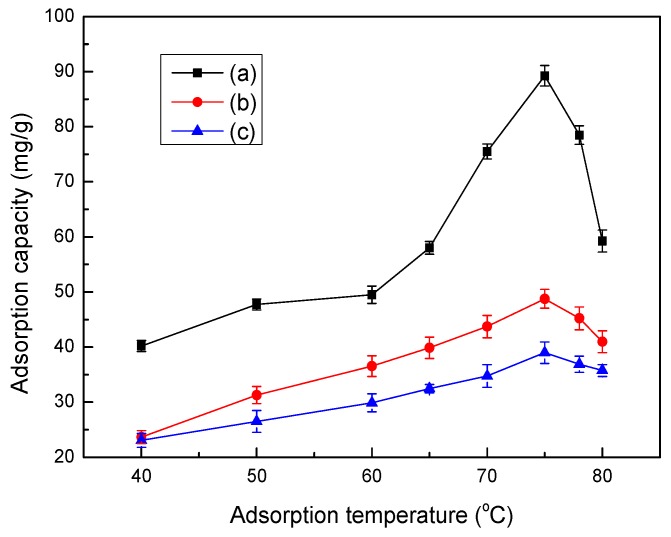
Effect of temperature on adsorption capacity of LMT (**a**), MT (**b**), and LC (**c**). Adsorbent: 0.1000 g; initial Co(II) concentration: 0.0040 mol/L; pH: 5.6; and time: 60 min.

The relationship between adsorption capacity and adsorption temperature is shown in Figure 7. The adsorption capacities of Co(II) first increased, and then decreased with increasing temperature. This result can likely be attributed to the enhanced activity of LMT molecules with an increase in adsorption temperature, caused by the disruption of intermolecular hydrogen bonding interactions between the molecular chains due to the acceleration of molecular thermal motion. With an increased number of activated molecules, the interaction between Co(II) and LMT was also enhanced, which was conducive to increasing the absorption capacity. In addition, increasing the temperature is known to increase the rate of diffusion of the adsorbate molecules across the external boundary layer and the internal pores of LMT, due to the decrease in the viscosity of the solution for highly concentrated suspensions. However, continued heating was shown to lead to the decomposition of LMT with damage to the three-dimensional (3D) structures. Higher temperatures have been found to be advantageous for adsorption, and that adsorption was an endothermic reaction [35]. Therefore, 75 °C was set as the temperature to optimize the other factors.

### 3.9. Effect of Time on Adsorption

**Figure 8:**
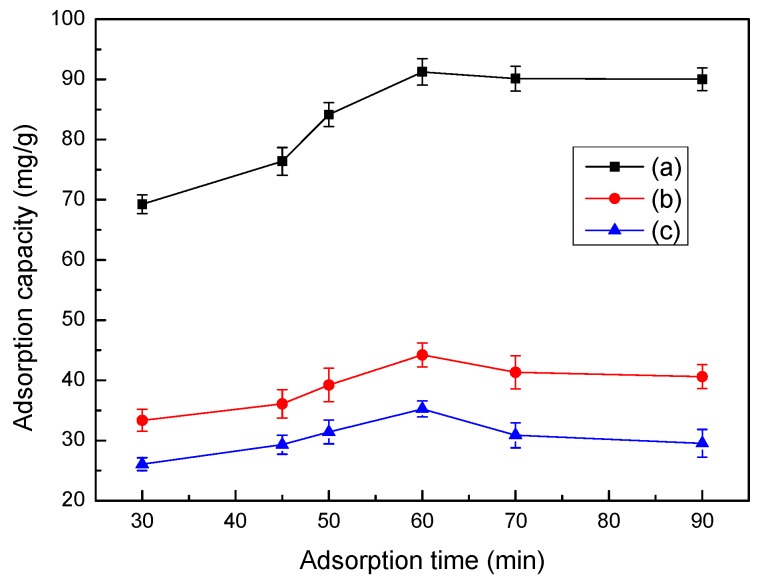
Effect of time on adsorption capacity LMT (**a**), MT (**b**), and LC (**c**). Adsorbent: 0.1000 g; initial Co(II) concentration: 0.0040 mol/L; pH: 5.6; and temperature: 75 °C.

The effects of adsorption time on the LMT nanocomposite adsorption capacity of Co(II) are shown in Figure 8. Initially, at prolonged adsorption times, the adsorption capacity of the LMT nanocomposite of Co(II) rapidly increased and then remained constant. This may be due to Co(II) being introduced to the adsorbent surface for a short contact time, which was then followed by a spread into the adsorbent micropores, finally forming a complex with the active sites of the adsorbent, resulting in a sharp adsorption equilibrium, so the adsorption capacity stayed constant [36]. Similarly, the optimum adsorption time of 60 min was selected for all experiments.

### 3.10. Kinetic Studies

**Figure 9:**
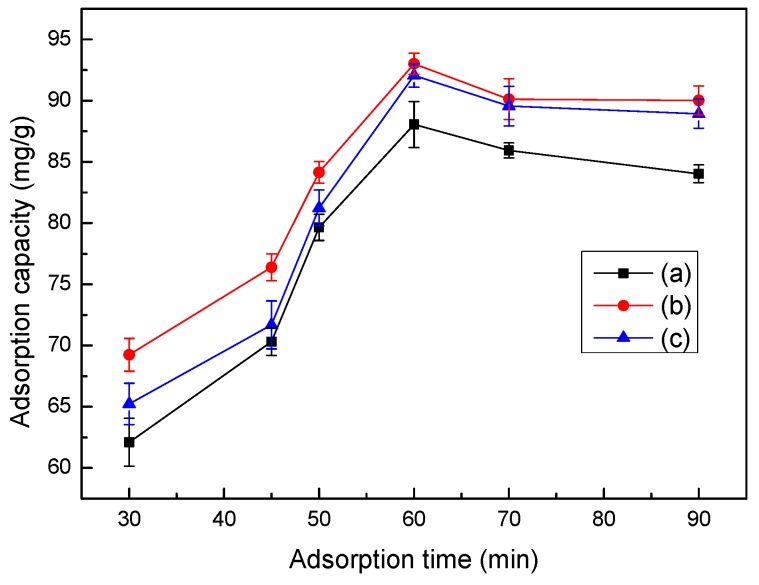
Effects of adsorption time on adsorption by LMT at various initial Co(II) concentrations of (**a**) 0.0035 M, (**b**) 0.0040 M, and (**c**) 0.0042 M.

The adsorption rate of LMT nanocomposite of Co(II) was investigated by assuming pseudo-first-order and pseudo-second-order models. Figure 9 shows the effect of adsorption time on the adsorption capacity of the LMT nanocomposite at various initial Co(II) concentrations. The *q_t_* increased significantly with increasing time *t* in minutes at the early stage and slowly dropped afterward. The maximum adsorption capacity of 93.02 mg/g was found at 60 min. We concluded that as the majority of the LMT nanocomposite adsorption sites (or pockets) remain, the adsorption rate is faster.

The adsorption kinetic curve of the LMT nanocomposite was modeled by fitting the pseudo-first-order adsorption kinetic data into Equation (3) and fitting the pseudo-second-order adsorption kinetic data into Equation (4) [37]:

(3) $$\log(q_{e}-q_{t})=\log q_{e}-\frac{k_{1}t}{2.303}$$

(4) $$\frac{t}{q_{t}}=\frac{1}{k_{2}q_{e}{}^{2}}+\frac{t}{q_{e}}$$

where *q_e_* (mg/g) is the amount of adsorption at equilibrium, *q_t_* (mg/g) is the adsorption amount at time *t* (min), *k*_1_ (min^−1^) is the rate constant of the pseudo-first-order adsorption kinetic equation, and *k*_2_ (g/(mg/min)) is the rate constant of the pseudo-second-order adsorption kinetic equation.

**Figure 10:**
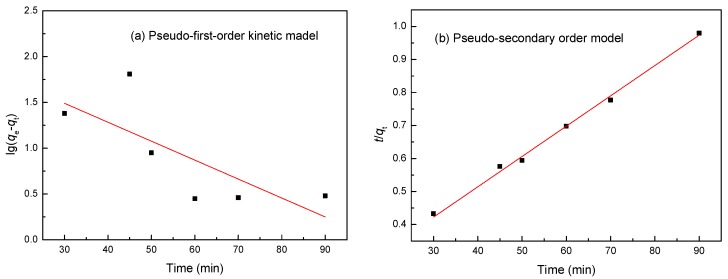
(**a**) Pseudo-first-order; and (**b**) pseudo-secondary-order adsorption kinetic equation fitting curves of the experimental data at a temperature of 75 °C, an initial Co(II) concentration of 0.0040 mol/L, and a pH of 5.6.

The results of adsorption kinetics are tabulated in Table 2, and the fitting models are shown in Figure 10. According to Figure 10 and Table 2, comparing the experimental equilibrium adsorption capacity for the adsorption of Co(II) onto the LMT nanocomposite, the results suggested an ideal fit with the pseudo-second-order model with an extremely high *R*^2^ of 0.9995. Good agreement was further supported by the similar values of the calculated and experimental values of *q_e__c_*. Therefore, the chemical adsorption should be the rate-limiting step for the adsorption of Co(II) onto the LMT nanocomposite [38].

### 3.11. Isotherm Studies

**Figure 11:**
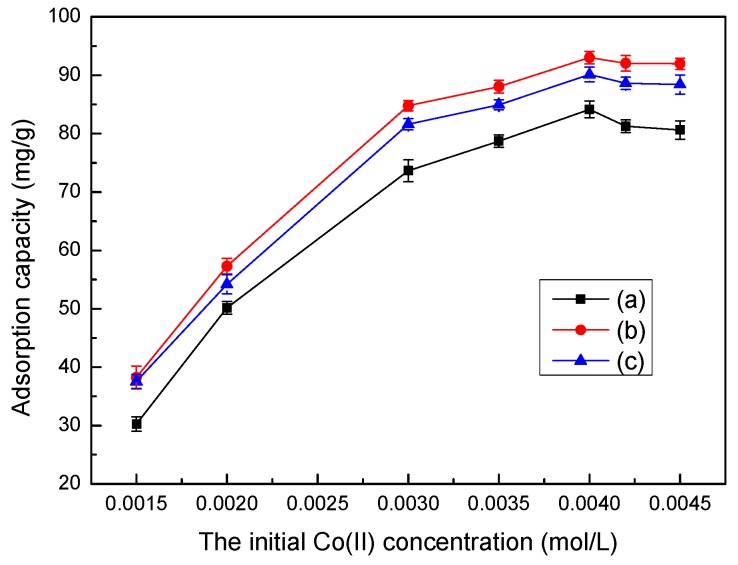
Effects of the initial Co(II) concentration on adsorption by LMT at various temperatures: 70 °C, 75 °C, and 78 °C.

An adsorption isotherm describes the equilibrium of the sorption of a material on the surface of an adsorbent. In this study, two adsorption isotherms, the Langmuir and Freundlich adsorption isotherm models, were used to interpret experimental data. Figure 11 shows the adsorption capacity of the LMT nanocomposite at different initial Co(II) concentrations at adsorption temperatures of 70 °C, 75 °C, and 78 °C. Isothermal adsorption curves were plotted based on the equilibrium adsorption amount *q_e_* and adsorption equilibrium concentration *C_e_*. In Figure 11, the adsorption equilibrium amount on the LMT nanocomposite surface was enhanced with increasing initial Co(II) concentration. Moreover, the degree of increase was higher at a lower concentration and decreased somewhat with increasing initial Co(II) concentration. At a low initial Co(II) concentrations, the LMT had enough active adsorption sites to interact with Co(II) ions; however, at higher initial Co(II) concentrations, the active adsorption sites were mostly occupied by Co(II) ions, limiting the adsorption.

Isothermal adsorption curves were fitted and plotted by employing the Langmuir and Freundlich equations, shown in Equations (5) and (6), respectively [39]:

(5) $$\frac{C_{e}}{q_{e}}=\frac{1}{K_{L}q_{\max}}+\frac{C_{e}}{q_{\max}}$$

(6) $$\ln q_{e}=\ln K_{f}+\frac{1}{n}\ln C_{e}$$

where *b* (L/mg) is the Langmuir constant relating to the adsorption capacity; *n* and *k_f_* are Freundlich constants; *C_e_* (mol/L) is the concentration of Co(II) at equilibrium; *q*_max_ (mg/g) is the monolayer saturation adsorption; and *q_e_* (mg/g) is the adsorption capacity at equilibrium.

The essential characteristics of the Langmuir isotherm can be represented according to a dimensionless equilibrium parameter (*R_L_*) based on the following equation [39]:

(7) $$R_{L}=\frac{1}{1+K_{L}C_{0}}$$

where *K_L_* (L/mg) is the Langmuir adsorption constant and *C*_0_ is the optimal concentration of Co(II) ions. The *R_L_* value indicates the nature of the isotherm is unfavorable (*R_L_* > 1), linear (*R_L_* = 1), favorable (0 < *R_L_* < 1), or irreversible (*R_L_* = 0).

**Figure 12:**
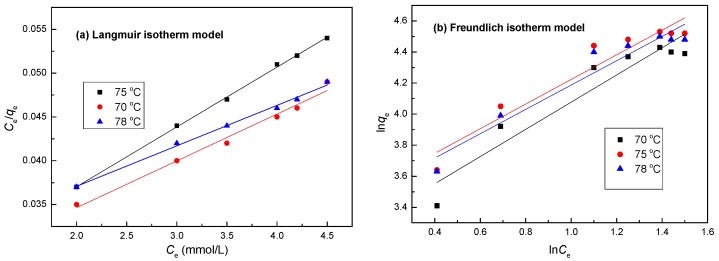
Adsorption isotherms of Co(II) onto LMT nanocomposite at different temperatures: (**a**) Langmuir; and (**b**) Freundlich.

The result of the isotherm equation and the fitting curves are shown in Table 3 and Figure 12, respectively. Based on the *R*^2^ value and fitting curve, the adsorption of Co(II) ions by the LMT nanocomposite falls into the Langmuir isothermal adsorption model, indicating that the mechanism involved is monolayer adsorption [40].

Comparisons of the isotherm models for the adsorption of Co(II) onto LMT were performed by comparing each linear plot of *C_e_*/*q_e_* versus *C_e_* (Figure 12a), and ln*q_e_* versus ln*C_e_* (Figure 12b). The calculated constants are listed in Table 3. From Figure 12 and Table 2, the *R*^2^ coefficients of the linear form of the Langmuir model, at 0.9987, were closer to 1 than that of the Freundlich model. In addition, the maximum monolayer adsorption capacity (*q*_max_) value calculated from the Langmuir model was 93.43 mg/g, which was almost the same as found in the experimental data, 93.02 mg/g. This result may be due to the homogeneous distribution of the activated sites on the surface of the LMT. Furthermore, the value of *R_L_* for the Langmuir isotherm was between 0 and 1, indicating a favorable process. Obviously, the Langmuir model more accurately described the adsorption of Co(II) onto LMT. The Langmuir model corresponds to a dominant electrostatic attraction, ion exchange, and coordination mechanism. This means that the adsorption process involved physical adsorption and monolayer coverage chemical complexation at the interface and the outer heterogeneous surface of the LMT nanocomposite.

### 3.12. Effect of Desorption Eluents on Desorption

**Figure 13:**
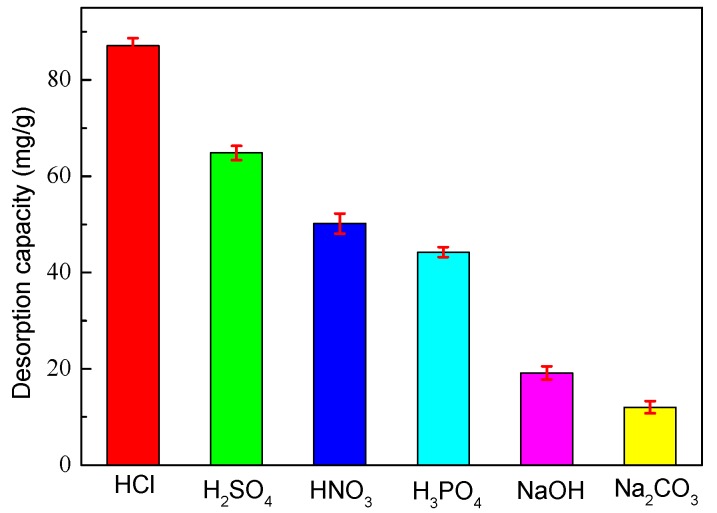
Effect of different desorption eluents on desorption capacity of LMT.

An important characteristic of the LMT nanocomposite is the possibility of its regeneration for further use. Repeated adsorption/desorption cycles experiments were conducted in the present work using HNO_3_, HCl, H_2_SO_4_, H_3_PO_4_, NaOH and Na_2_CO_3_, separately, as the desorbing eluents to investigate their effects on desorption (Figure 13). Figure 13 clearly indicates that NaOH and Na_2_CO_3_ are almost ineffective at releasing bonded Co(II) ions from LMT nanocomposite. The desorption capacity of NaOH was slightly higher than Na_2_CO_3_, but still showed lower desorption capacity when compared with the acid eluents. Among the four acidic desorption eluents, HNO_3_ was found to be the most appropriate desorption eluent for the regeneration of Co(II)-loaded LMT. This result was expected because the surface of LMT was protonated by H^+^ ions under acidic conditions, and electrostatic interactions occurred between H^+^ and the activated sites of the surface of LMT, thereby allowing the desorption of positively charged Co(II). Furthermore, ion-exchange, electrostatic attraction and coordination mechanisms were determined, and HNO_3_ could be an effective desorption eluent for the regeneration of Co(II)-loaded LMT in this work.

### 3.13. Effect of HNO_3_ Concentration on Desorption

**Figure 14:**
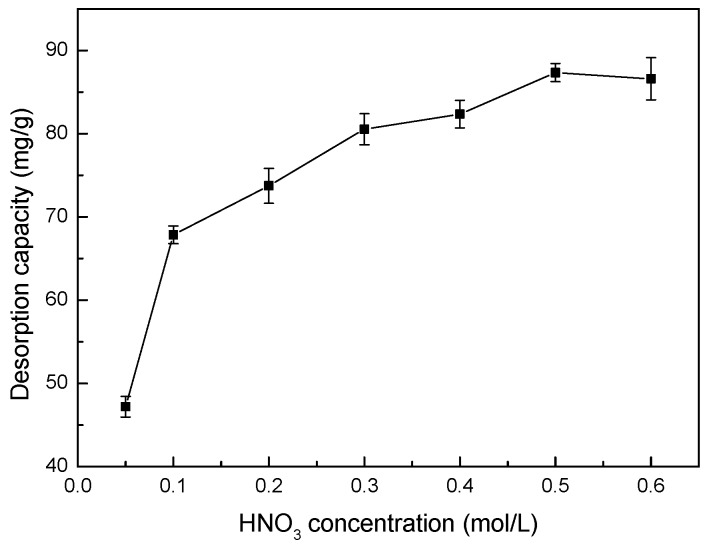
Effect of nitric acid (HNO_3_) concentration on desorption capacity of LMT.

The effects of different HNO_3_ concentrations on the desorption capacity of Co(II)-loaded LMT are shown in Figure 14. The desorption capacity of the LMT nanocomposite first increased and then stayed constant with increasing HNO_3_ concentration.

The concentrated H^+^ appeared to be the driving force for desorption of Co(II), to a limit, by positive ion exchange and increased the concentration gradients of Co(II) and H^+^, further facilitating the desorption of loaded-Co(II). However, high concentrations of H^+^ beyond this limit would not increase the desorption capacity, similar to a report in the literature [41]. The relatively high desorption capacity of the LMT nanocomposite reached 87.34 mg/g at a HNO_3_ concentration of 0.5 mol/L, suggesting that the adsorption of Co(II) onto LMT partially occurred via electrostatic attraction and ion exchange, substantiating the results for pH values with respect to adsorption.

### 3.14. Effect of Temperature on Desorption

**Figure 15:**
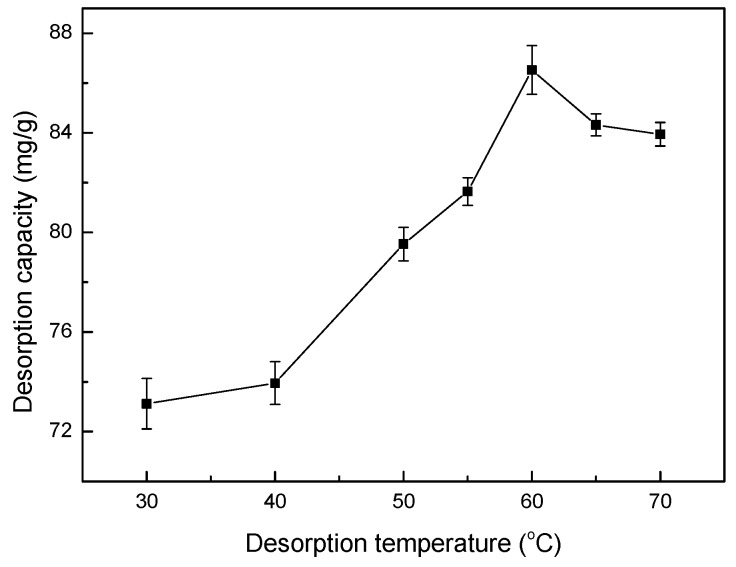
Effect of temperature on desorption capacity of LMT. Co(II)-loaded LMT nanocomposite: 0.1000 g; nitric acid (HNO_3_) concentration: 0.5 mol/L; and time: 55 min.

The effects of different desorption temperatures on the desorption capacity of Co(II)-loaded LMT nanocomposite are shown in Figure 15. The desorption capacity of the LMT nanocomposite first increased and then decreased with increasing temperature. This fluctuation could be attributed to the fact that increasing temperature may enhance the adsorption activity and efficiency of the activated sites on the surface of the LMT. H^+^ and Co(II) may compete for the activated sites, leading to an increase in desorption efficiency. In addition, a higher desorption temperature may impair the adsorption efficiency of the active sites and lead to the decomposition of LMT with damage to the 3D structures, having a detrimental effect on the desorption process. This further supports the results for adsorption temperature [42,43]. Therefore, 60 °C was used in all experiments.

### 3.15. Effect of Sonication Time on Desorption

**Figure 16:**
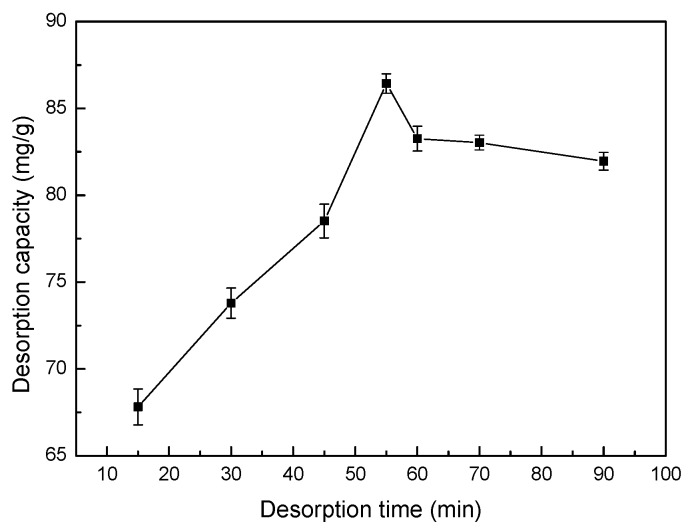
Effect of time on desorption capacity of LMT. Co(II)-loaded LMT nanocomposite: 0.1000 g; HNO_3_ concentration: 0.5 mol/L; and temperature: 60 °C.

The effects of different desorption times on the Co(II)-loaded LMT nanocomposite are shown in Figure 16. The desorption capacity of the LMT nanocomposite first increased and then decreased with increase in desorption time. This phenomenon was consistent with that of kaolinite [44]. The LMT nanocomposite reached a maximum desorption at 55 min of desorption time.

### 3.16. Recycling and Reusability of LMT

Recovering and reusing an adsorbent would decrease the processing cost, and is an important parameter for practical applications [45]. The reusability of the LMT was tested with five consecutive adsorption/desorption cycles. All adsorption and desorption capacities of LMT nanocomposite in the five consecutive cycles are tabulated in Table 4. The data showed that the LMT could be recycled up to four times without significantly losing its adsorption/desorption capacities. After the fourth cycle, the adsorption and desorption capacities were 80.07 mg/g and 68.43 mg/g, respectively, showing that the LMT nanocomposite is a good reusable adsorbent for the removal of Co(II).

### 3.17. Adsorption Mechanism of Co(II)

**Figure 17:**
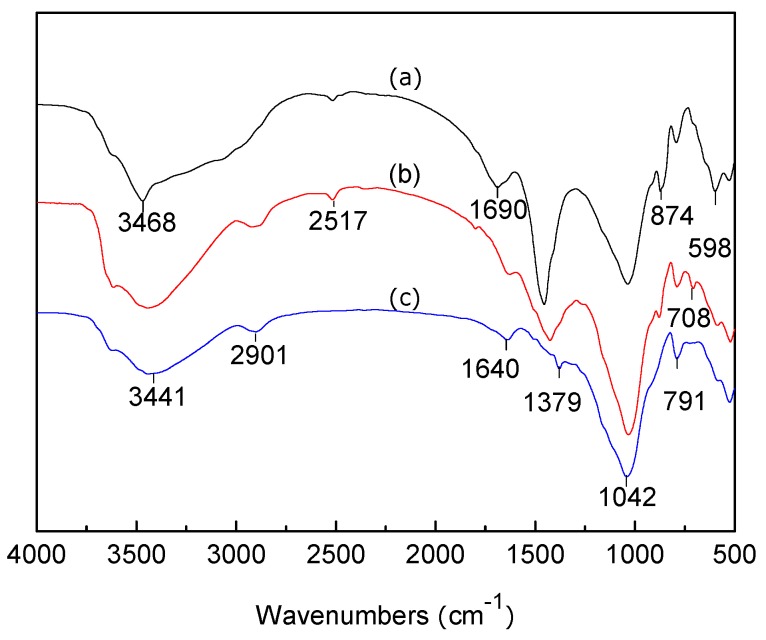
Fourier Transform Infrared Spectroscopy (FTIR) spectra of (**a**) LMT, (**b**) Co(II)-loaded LMT, and (**c**) recovered LMT.

Fourier Transform Infrared Spectroscopy (FTIR) spectra of pure LMT, Co(II)-loaded LMT, and recovered LMT are shown in Figure 17. The adsorption bands at 3468 cm^−1^ (Figure 17a) are attributed to the intramolecular O–H stretching vibration absorption peak, as well as the characteristic absorption band of intermolecular hydrogen bonding between phenol and alcohol molecules. The band shifted to a lower wavenumber at 3441 cm^−1^ (Figure 17b) after the adsorption of Co(II), indicating that some of the O–H and corresponding hydrogen bonds interacted with Co(II), and this band weakened after desorption (Figure 17c). The characteristic adsorption band at 1690 cm^−1^ (Figure 17a), corresponding to the asymmetric stretch vibration of the C=O bond in carboxylic acids, disappeared (Figure 17b) after adsorption of Co(II). It appeared again after desorption of Co(II) at a lower wavelength 1640 cm^−1^ (Figure 17c). The vibration absorption peak of the carboxyl O–H bond, located at 1402 cm^−1^ (Figure 17a), disappeared after Co(II) adsorption, reappearing at 1379 cm^−1^ (Figure 17c) after desorption of Co(II). Moreover, the absorption band at 874 cm^−1^ (Figure 17a), which represents the stretching vibration absorption of the aromatic and phenol C–H stretching bond, moved to a lower wavelength after Co(II) absorption, and then shifted back down to 791 cm^−1^ (Figure 17c) after desorption. Based on the above-mentioned results, it was tentatively concluded that protons of the hydroxyl and carboxyl functional groups of LMT were replaced by Co(II) and the free carboxyl groups became carboxylates after adsorption. Generally, ion exchange occurred, and chemical bonds were formed between Co(II) and the –OH and –COOH groups of LMT nanocomposite. Moreover, slight changes were observed in the FTIR spectra of Co(II)-loaded LMT nanocomposite, and they were basically restored to their original shape after desorption. Overall, the basic structure and properties of the LMT remained relatively stable in the process of Co(II) adsorption and desorption, showing that it is a good renewable adsorbent.

**Figure 18:**
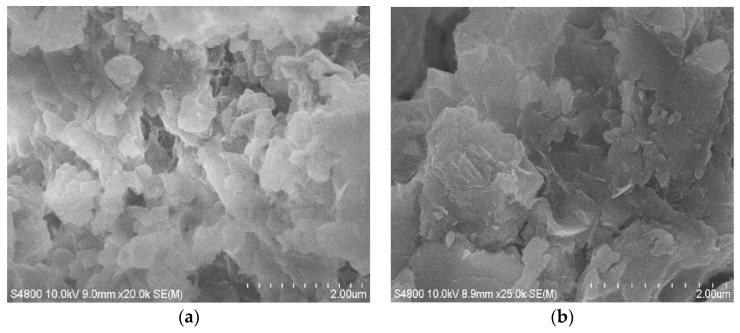
Scanning Electron Microscope (SEM) images of: (**a**) Co(II)-loaded LMT; and (**b**) recovered LMT.

The Scanning Electron Microscope (SEM) images of LMT, Co(II)-loaded LMT, and recovered LMT are shown in Figure 3b,c and Figure 18, respectively. Figure 3b,c displays a coarse, rough surface of pure LMT nanocomposite with the presence of pores and cavities, whereas, after adsorption of Co(II), Co(II)-loaded LMT (Figure 18a) showed a lamellar curly surface, which was evenly packed with Co(II) ions and the sheet-stacking structure disappeared. A non-uniform distribution of adsorbed Co(II) was observed at the adsorption surface sites of LMT, and most of the mesopores at the surface were completely filled with Co(II) ions. Some regions exhibited higher concentrations of Co(II) than others. This suggested the formation of a uniform adsorption layer on the LMT surface [46]. Moreover, the recovered LMT (Figure 18b) showed the irregular clusters of polymerized sheet-stacking dispersion on the surface. These results indicated that the adsorption of LMT of Co(II) may be a chemical interaction; in addition, considering the presence of sparse and preferential adsorption sites on the surface of LMT, physical porous adsorption is also involved, which support the previously proposed adsorption mechanism.

**Figure 19:**
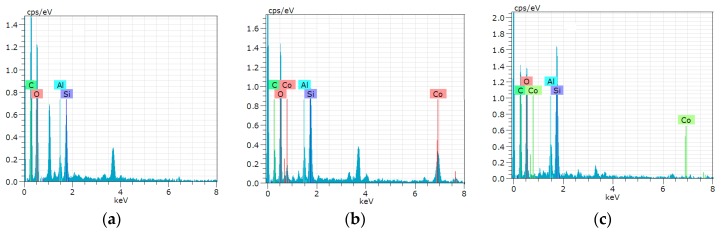
Energy-Dispersive X-ray (EDX) spectra for: (**a**) LMT; (**b**) Co(II)-loaded LMT; and (**c**) recovered LMT.

The Energy-Dispersive X-ray (EDX) is an analytical technique used for elemental analysis. EDX analysis of LMT was performed to confirm the existence of Co(II) on Co(II)-loaded LMT. EDX spectra of pure LMT (Figure 19a), Co(II)-loaded LMT (Figure 19b), and recovered LMT (Figure 19c) are shown. In the EDX spectrum, two new Co(II) peaks were found in Co(II)-loaded LMT, confirming the presence of Co(II) ions (Figure 19b). After desorption, content of Co(II) decreased (Figure 19c).

### 3.18. Comparison with Previously Reported Data for Co(II) Adsorption

The values of adsorption capacity by the other adsorbents from the literature are given in Table 5 for comparison. Through the comparative study, we can conclude that LMT is one of the most powerful adsorbents prepared for Co(II) ions.

## 4. Conclusions

A reusable LMT nanocomposite was prepared using a chemical intercalation reaction. The LMT nanocomposite was characterized by N_2_ adsorption/desorption isotherm, FTIR, XRD, SEM, and TEM. The results demonstrated that the slender and scattered LC was dispersed into the MT nanoplatelets, and an intercalated-exfoliated nanostructure was formed in the LMT, displaying a higher surface area of 245.1 m^2^/g and total pore volume of 1.692 cm^3^/g, greatly improving the nano-effect. LMT can be effectively applied for the adsorption of Co(II) ions from aqueous solutions. The maximum adsorption capacity, with 0.10 g of LMT for Co(II), reached 93.02 mg/g under optimal conditions of an initial Co(II) concentration of 0.0040 mol/L, a pH of 5.6, an adsorption temperature of 75 °C, and an adsorption time of 60 min. The adsorption kinetics and isotherms were well-fitted to both the pseudo-second-order adsorption kinetics equation, with an *R*^2^ value of 0.9992, and the Langmuir isothermal adsorption model, with an *R*^2^ of 0.9987. These results indicate that the adsorption equilibrium was mainly dominated by monolayer chemical adsorption in the experimental range.

The effects on the desorption capacity of the Co(II)-loaded-LMT were observed by using HNO_3_ as a desorption agent in the ultrasonic oscillation treatment. The optimum conditions of desorption were as follows: the concentration of HNO_3_ was 0.5 mol/L, the desorption temperature was 60 °C, and the time for ultrasonic desorption was 55 min. Under the optimum conditions, the maximum desorption capacity was determined as 87.34 mg/g.

The adsorption/desorption experiments demonstrated that the adsorption, desorption capacity, and desorption efficiency of LMT remained at a relatively high level after four rounds of adsorption/desorption recycling. This study showed that the LMT nanocomposite is an excellent and renewable potential adsorbent for the removal of Co(II) ions from aqueous solutions.

## Figures and Tables

**Table 1:** Pore structure parameters of MT, LMT nanocomposite and Co(II)-loaded LMT used in this study.

Sample	*S*_BET_ (m^2^/g)	*S*_ext_ (m^2^/g)	*S*_ext_/*S*_BET_ (%)	*S*_mic_ (m^2^/g)	*V*_tot_ (cm^3^/g)	*V*_meso_ (cm^3^/g)	*V*_meso_/*V*_tot_ (%)	*D*_p_ (nm)
MT	87.6	50.2	57.3	19.1	1.013	0.651	64.3	103.8
LMT	245.1	177.4	73.4	52.0	1.692	1.175	69.4	57.7
Co(II)-loaded LMT	112.3	46.2	41.1	29.4	1.117	0.395	35.4	30.6

*S*_BET_: specific surface area; *S*_ext_: mesopore surface area; *S*_ext_/*S*_BET_: ratio of mesopore surface area to specific surface area; *S*_mic_: micropore surface area; *V*_tot_: total pore volume; *V*_meso_: mesopore volume; *V*_meso_/*V*_tot_: ratio of mesopore volume to total pore volume; and *D*_p_: average pore size. Number of replicates: 3.

**Table 2:** Kinetic parameters for Co(II) adsorption onto LMT.

Metal	Parameter	Pseudo-First-Order		Pseudo-Second-Order	
**Co(II)**	*R*^2^	0.4667		0.9995	
	Constants	*k*_1_	0.0013 min^−1^	*k*_2_	0.0001 min^−1^
		*q_ec_*	78.15 mg/g	*q_ec_*	92.98 mg/g
		*q_e_*	93.02 mg/g	*q_e_*	93.02 mg/g

**Table 3:** Isotherm parameters for Co(II) adsorption onto LMT at different temperatures.

Isotherm	Parameter	Temperature (°C)		
		70	75	78
**Langmuir Model**	*q*_max_ (mg/g)	87.14	93.43	91.60
	*K_L_* (L/mg)	0.0017	0.0065	0.0043
	*R_L_*	0.84	1.06	0.62
	*R*^2^	0.9543	0.9987	0.9702
**Freundlich Model**	*K_f_* (mg g^−1^ (L mg^−1^)1/n)	2.8	9.7	3.5
	*n*	1.05	1.87	1.33
	*R*^2^	0.8903	0.9080	0.9201

**Table 4:** LMT adsorption/desorption capacities for Co(II) in five consecutive cycles.

Cycle Number	1st	2nd	3rd	4th	5th
**Adsorption *q_e_* (mg/g)**	93.02	90.51	85.44	80.07	51.17
**Desorption *q_e_* (mg/g)**	87.34	81.64	78.46	68.43	37.26

**Table 5:** Comparison of Co(II) adsorption capacity by various adsorbents from the literatures.

Adsorbent Material	Adsorption Capacity (mg/g)	Reference
LMT	93.02	This study
Cuttlefish bones	76.76	[47]
Almond green hull	45.5	[48]
CIF-BC	45.44	[49]
ZrO-Montmorillite	22.8	[50]
Red mud waste material	18.05	[51]
Natural zeolites	14.38	[52]
